# A Convenient Fluorescence-Based Assay for the Detection of Sucrose Transport and the Introduction of a Sucrose Transporter from Potato into *Clostridium* Strains

**DOI:** 10.3390/molecules24193495

**Published:** 2019-09-26

**Authors:** Zhikai Zhang, Lihua Lin, Hongchi Tang, Shaowei Zeng, Yuan Guo, Yutuo Wei, Ribo Huang, Hao Pang, Liqin Du

**Affiliations:** 1 State Key Laboratory for Conservation and Utilization of Subtropical Agro-bioresources, Guangxi Research Center for Microbial and Enzymatic Technology, College of Life Science and Technology, Guangxi University, Daxue Road No. 100, Nanning 530005, China; zhikaizhang10@163.com (Z.Z.); guruace@163.com (R.H.); 2 National Engineering Research Center for Non-Food Biorefinery, State Key Laboratory of Non-Food Biomass and Enzyme Technology, Guangxi Key Laboratory of Bio-refinery, Guangxi Academy of Sciences, Daling Road No. 98, Nanning 530007, China; linlihua_8@126.com (L.L.); panghouse@126.com (H.P.)

**Keywords:** sucrose transporter, esculin, fluorescence assay, *Clostridium*

## Abstract

A convenient and effective sucrose transport assay for *Clostridium* strains is needed. Traditional methods, such as ^14^C-sucrose isotope labelling, use radioactive materials and are not convenient for many laboratories. Here, a sucrose transporter from potato was introduced into *Clostridium*, and a fluorescence assay based on esculin was used for the analysis of sucrose transport in *Clostridium* strains. This showed that the heterologously expressed potato sucrose transporter is functional in *Clostridium*. Recombinant engineering of high-level sucrose transport would aid sucrose fermentation in *Clostridium* strains. The assay described herein provides an important technological platform for studying sucrose transporter function following heterologous expression in *Clostridium*.

## 1. Introduction

It is of current interest to determine methods to efficiently use sugars (such as cellulose, xylose, and sucrose) from non-food biomass feedstocks as the carbon source for microbial fermentation [1,2,3], as this is a key factor restricting the conversion of non-food biomass to biofuel and high value-added chemicals. Sugar transport systems may play an important role in this process [4,5]. Genetic engineering at the transporter level is an evolving field; however, there have been few studies focused on improving the yield of biofuels. In contrast, much research has been performed on yeast [6,7,8], and the latest reports show that engineering of the yeast sugar transport system enhances fermentation [9,10,11]. Hyperthermophiles are interesting bacteria that encode many ATP binding cassette type (ABC-type) transporters and recently these transporters have been used to increase hydrogen production. The maltose transporter was genetically characterized in *Thermotoga maritima*, and genetic engineering of this transporter improved the fermentation efficiency of this bacterium [12,13]. Introduction of a sucrose permease from *Escherichia coli* and a sucrose phosphorylase from *Streptococcus mutans* into *Bacillus subtilis* resulted in a recombinant strain that showed remarkable enhancement of 2,3-butanediol production compared with the control strain [14]. Overexpression of an endogenous sucrose utilization system, the phosphoenolpyruvate-phosphotransferase system (PEP-PTS) transport protein for sucrose and the related sucrose hydrolase enzymes elevated sucrose consumption and enhanced acetone-butanol-ethanol (ABE) production in *Clostridium saccharoperbutylacetonicum* N1-4 [15].

Sucrose is the most abundant disaccharide in the environment and an important feedstock for the fermentation industry. In bacteria, there are two pathways to transport sucrose: the phosphoenolpyruvate-phosphotransferase system, which results in the accumulation of sucrose-6-phosphate, and the sucrose permease transport system, which transports unmodified sucrose into the cell. In plants, sucrose transporters (SUTs) operate by the cellular transport of proton-coupled sucrose [16,17,18,19]. Type I and II SUTs are localized in the plasma membrane, while type III SUTs are localized in the vacuole membrane [17,19]. Several different methods have been employed to detect the activity of SUTs. ^14^C-sucrose isotope labelling was the first method reported for analyzing sucrose uptake and transport in plants [16]. SUTs from plants were cloned and heterologously expressed in yeast, and sucrose uptake was analyzed using ^14^C-sucrose isotope labelling methods [6], which indicated that yeast was an ideal model in which to study the function of SUTs.

More recently, to rapidly and easily detect sucrose transport activity in yeast, a novel method was developed based on the ability of type I SUTs to transport esculin (6,7-dihydroxycoumarin 6-glucoside, 7-hydroxy-6-[(2S,3R,4S,5S,6R)-3,4,5-trihydroxy-6-(hydroxymethyl)oxan-2-yl]oxychromen-2-one) [16]. Esculin is a structural analogue of sucrose with strong fluorescence (excitation 367 nm, emission 454 nm), and the fluorescence positively correlates with the amount of esculin taken up into the cells. Compared with radioactive carbon, the use of esculin is safer and readily available in most labs. 

The sucrose transporter StSUT1 from potato (*Solanum tuberosum*) was expressed in *Saccharomyces cerevisiae* and was found to be a type I SUT [20]. Butanol is a renewable biofuel that can be produced by fermentation in *Clostridium* [21,22], but the ability of *Clostridium* to use sucrose is low [23,24]. Recombinant engineering of *Clostridium* to achieve high levels of sucrose transport would aid sucrose consumption in *Clostridium* to produce butanol [15]. In this work, we show that heterologously expressed StSUT1 was functional in *Clostridium. beijerinckii* and *Clostridium. acetobutylicum,* and transport of sucrose through this transporter was confirmed by an esculin fluorescence-based method.

## 2. Results

### 2.1. Heterologous Expression of the StSUT1 Gene in C. beijerinckii and C. acetobutylicum 

**Figure 1:**
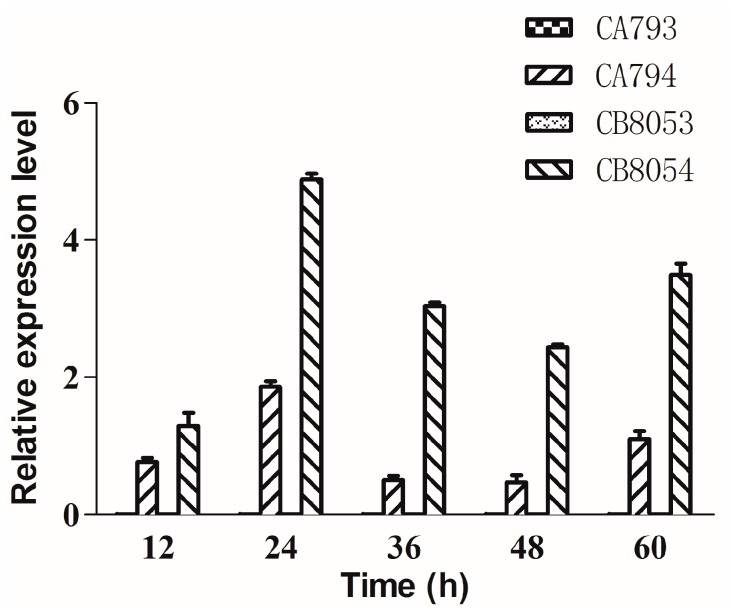
The expression of *StSUT1* analyzed by quantitative real time polymerase chain reaction. Cells were collected at 12, 24, 36, 48, and 60 h. Three independent biological replicates were performed for each gene. The data represent the means of three separate experiments ± standard deviation. The control strains CA793 and CB8053 did not express *StSUT1*.

The type I sucrose transporter StSUT1 from potato was overexpressed in *C. beijerinckii* 8052 (designated strain CB8054) and *C. acetobutylicum* 792 (designated strain CA794). The gene expression level of *StSUT1* was measured by real-time quantitative PCR analysis (qRT-PCR). The results suggested that the *StSUT1* gene was effectively expressed in both *Clostridium* strains, whereas the *StSUT1* gene transcript was not detected in the control strains (designated strains CA793 and CB8053) (Figure 1). The relative expression level of *StSUT1* in strain CB8054 was significantly higher than that in strain CA794. A *t*-test was applied for difference comparison among the measured data and the P value was found to be 0.012. These results indicated that StSUT1 was more effectively expressed in *C. beijerinckii* compared with *C. acetobutylicum*, likely enhancing the use of sucrose by *C. beijerinckii*. 

### 2.2. Determination of the Activity of the Sucrose Transporter StSUT1 by a Fluorescence-Based Assay

**Figure 2:**
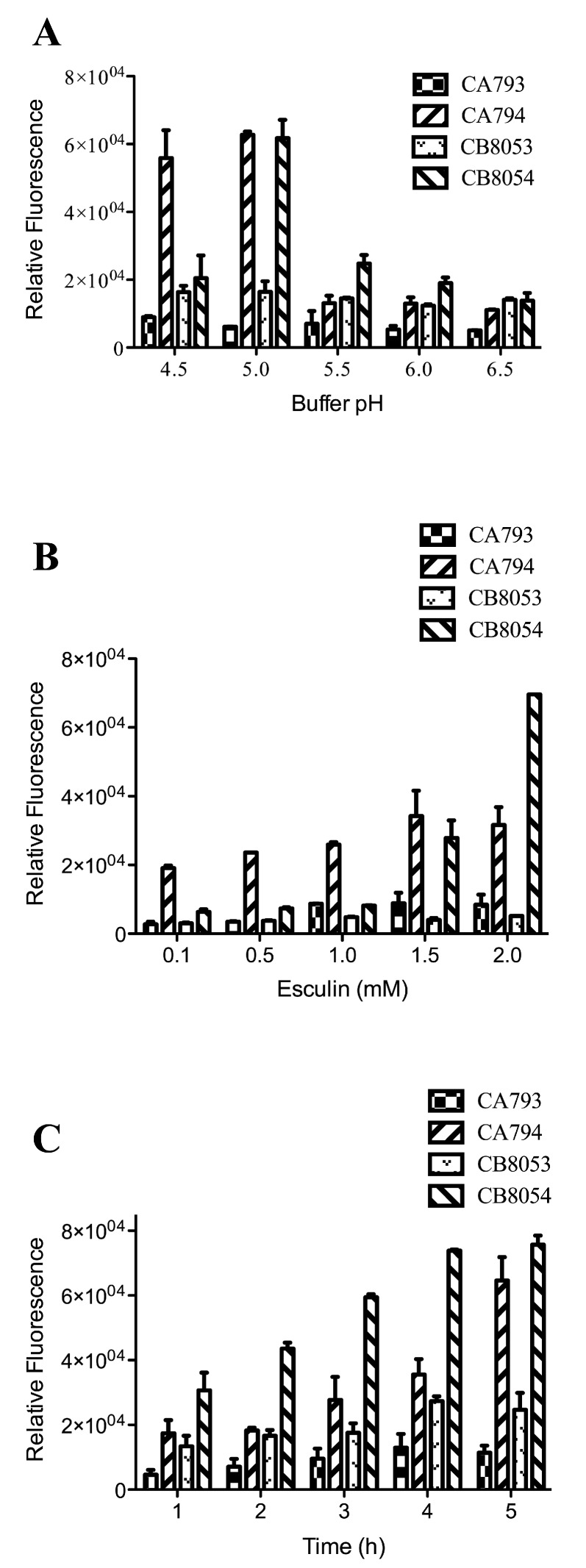
Analysis of esculin uptake in *Clostridium* strains under different conditions. (**A**) Effect of pH on esculin uptake. *Clostridium* cells were incubated with 1.0 mM esculin for 2 h at different pH values. (**B**) Effect of the concentration of esculin on esculin uptake. *Clostridium* cells were incubated with different concentrations of esculin at pH 5.0 for 2 h. (**C**) Effect of incubation time on esculin uptake. *Clostridium* cells were incubated with 1.0 mM esculin at pH 5.0 for the indicated times. The data represent the mean of three separate experiments ± standard deviation.

Strains CB8053, CB8054, CA793, and CA794 were incubated with 1 mM esculin at pH 4.5, 5.0, 5.5, 6.0, and 6.5 for 2 h. The strains containing StSUT1 emitted higher fluorescence than the vector controls except at pH 6.5 (Figure 2A). At pH 6.5, strain CB8054 showed the same fluorescence value as the control strain, indicating that the recombinant transport system did not work at this pH. At pH 5.0, strain CB8054 showed the highest fluorescence emission (61,750 ± 5,411) among all the tested pH conditions, and the fluorescence sharply decreased to 20,479 ± 6,674 when the pH was decreased to 4.5. Similarly, the fluorescence value for strain CA794 was highest at pH 5.0.

Strains were incubated with different concentrations of esculin. When incubated with 0.1, 0.5, 1.0, 1.5 and 2.0 mM esculin, the fluorescence generally increased with the esculin concentration (Figure 2B). For *C. acetobutylicum* at 0.1 mM esculin, there was an obvious fluorescence signal compared with the control strain; the relative fluorescence value was 19,069 ± 744 compared with 2,792 ± 688 for the control. The fluorescence increased up to 1.5 mM esculin. In *C. beijerinckii*, at low esculin concentration, the transport of esculin was limited; thus, the fluorescence value was only 6,335 ± 755 at 0.1 mM esculin. However, when the concentration of esculin was increased to 1.5 mM, the fluorescence was 27,851 ± 5,099, and at 2.0 mM esculin it reached 69,663 ± 0. A higher concentration of esculin was not tested because of its low solubility in water.

Strains were also incubated with esculin for different times. When strains CB8053, CB8054, CA793 and CA794 were incubated for 1 to 5 h in 1 mM esculin at pH 5.0, high fluorescence values were obtained that differed slightly among the strains (Figure 2C). After incubation for 1 h, strains CB8054 and CA794 showed an obvious difference in fluorescence compared with their respective control strains.

Taken together, these results show that the *Clostridium* strains express the type I SUT from potato, the activity of which can be rapidly detected using a fluorescence-based assay.

### 2.3. Sucrose Competition Effect on the Transport System 

**Figure 3:**
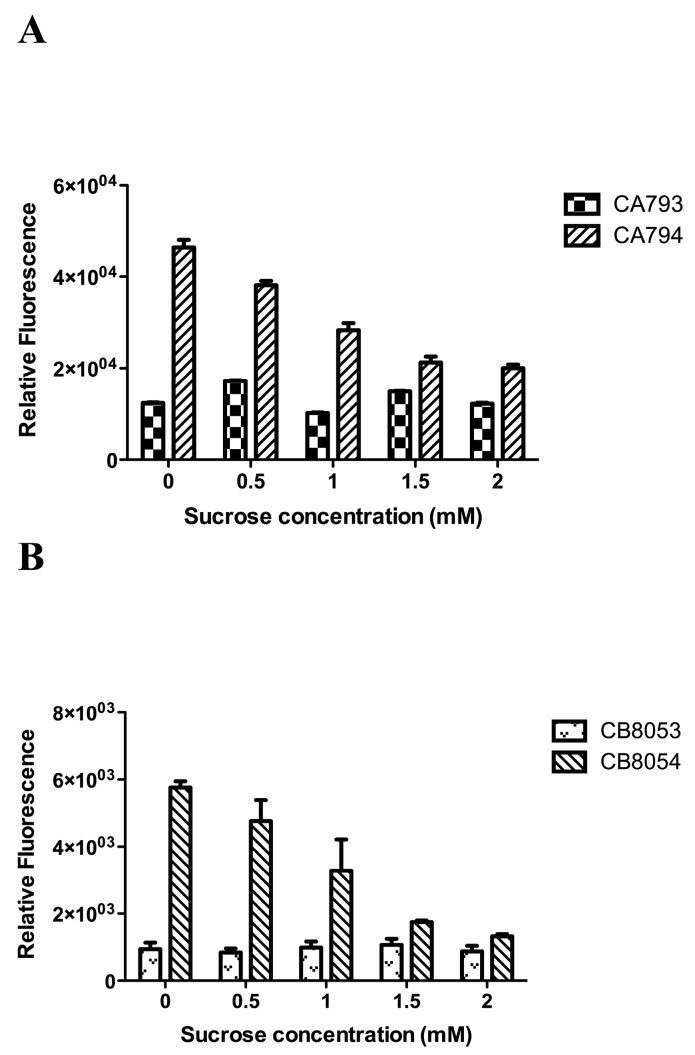
The competition effect of sucrose on esculin transport. *Clostridium* cells were incubated with 1.0 mM esculin for 2 h at pH 5.0. The data represent the mean of three separate experiments ± standard deviation. (**A**) Effect of sucrose on esculin uptake in *Clostridium acetobutylicum* strains. (**B**) Effect of sucrose on esculin uptake in *Clostridium beijerinckii* strains.

Strains were incubated with 1 mM esculin and different concentrations of sucrose. Sucrose showed an obvious inhibitory effect on esculin transport in strains CA794 and CB8054, but not in the control stains CA793 and CB8053 (Figure 3). Thus, sucrose competed with esculin for the StSUT1 transporter in strains CA794 and CB8054, indicating that sucrose and esculin use the same transport system in strains CA794 and CB8054.

## 3. Discussion

Renewable sugar, cellulose, and starch biomass are important substrates in industrial ABE fermentation. Sucrose is a cheap and ideal feedstock for ABE fermentation [15]. In *C. beijerinckii* 8052 and *C. acetobutylicum* DSM 792, ^14^C-sucrose isotope labelling was employed to demonstrate that sucrose transport occurs via a PEP-PTS mechanism [23,25,26,27]. In *C. beijerinckii*, both glucose and sucrose were initially metabolized at the same rate indicating that glucose regulates sucrose use at some level, but does not directly inhibit sucrose uptake [27]. In *C. acetobutylicum*, sucrose was needed to induce the expression of the PEP-PTS system; however, induction was prevented by the presence of glucose in the culture medium because glucose regulates the PTS gene system, which affected sucrose metabolism [23]. Although the PEP-PTS sucrose uptake system is conserved in both *C. beijerinckii* and *C. acetobutylicum*, the organization and regulatory elements of the operons for sucrose metabolism show considerable differences [23,28].

From our results, it is evident that the type I sucrose transporter StSUT1 from potato showed high esculin transfer ability in both *C. beijerinckii* and *C. acetobutylicum* strains (Figure 2 and Figure 3). Esculin, a structural analogue of sucrose, is a highly fluorescent molecule. Fluorescence is a type of photoluminescence that is the result of a molecule absorbing light at a specific wavelength and emitting light at a longer wavelength. For esculin, the absorbed light occurs at 367 nm and the emitted light occurs at 454 nm; the fluorescence positively correlates with the amount of esculin taken up into cells [20]. Thus, it is possible to measure the transport of esculin by a transport system. Moreover, it was found that esculin was transported at a rate similar to the transport of sucrose. Therefore, esculin was used as a tool to characterize the sucrose transport activity of the SUT1 transporter from potato when expressed in *Clostridium* strains [29]. 

Finding new metabolic pathways to increase sucrose consumption by *Clostridium* is one way to use this inexpensive and desirable substrate for ABE fermentation. Recently, Zhang et al. enhanced endogenous and introduced heterologous sucrose utilization pathways into *C. saccharoperbutylacetonicum* [15]. In their work, overexpression of the endogenous sucrose pathway increased sucrose consumption and ABE production. However, they failed to find heterologous sucrose utilization pathways that could compete with the native sucrose utilization pathways in the host strain. H^+^-coupled sucrose transporters from plants are considered to be the most efficient sucrose transport system [19,30,31]. StSUT1 from potato is an H^+^-coupled sucrose transporter and was able to transport esculin into *Clostridium* cells, and sucrose competition experiments showed that sucrose competed with esculin for transport in recombinant strains CA794 and CB8054 (Figure 3). The sucrose transporter StSUT1 was confirmed to actively uptake sucrose at acidic pH because it operates by a proton-coupled mechanism [30,31]. Environmental pH has been reported to be an important factor in the growth and fermentation of *Clostridium* [32,33]. In previous studies, it was demonstrated that pH values ranging from 5.5 to 6.0 were optimal for ABE fermentation [33,34]. However, for esculin uptake in the present study, the optimal pH was 5.0 (Figure 3A). At low pH, the sucrose transporter StSUT1 showed high activity, but this is not the best condition for growth and fermentation of *Clostridium*. Thus, further studies are needed to optimize conditions for the recombinant sucrose uptake system with simultaneous growth and fermentation of *Clostridium*.

In summary, our findings provide an important technological platform for studying sucrose transporter function by heterologous expression in *Clostridium*.

## 4. Materials and Methods 

### 4.1. Bacterial Strains and Plasmids

Two *Clostridium* strains were used in this study. The characteristics of these strains, *C. beijerinckii* NCIMB 8052 [35] and *C. acetobutylicum* DSM 792 (ATCC 824) [36], have been described in previous reports. *Escherichia coli* JM109 was used as the cloning host and was purchased from Invitrogen (Carlsbad, CA, USA). Plasmid pAN1 was used for methylating target plasmids [37] and plasmid pSOS95 was used as the expression vector in *Clostridium* strains [38]. The *StSUT1* open reading frame was cloned from plasmid pDR196-StSUT1, which was kindly gifted by Prof. John M. Ward [20].

### 4.2. Media and Culture Conditions

*Clostridium* strains and *E. coli* were stored as frozen cultures at −80 °C. *E. coli* was cultured at 37 °C in Luria-Bertani medium supplemented with 50 μg/mL ampicillin or 25 μg/mL chloramphenicol. *C. beijerinckii* NCIMB 8052 and *C. acetobutylicum* DSM 792 were cultured in Tryptone-yeast extract-acetate (TYA) medium (glucose 40 g/L, yeast extract 2 g/L, tryptone 6 g/L, beef extract 2 g/L, NH_4_Ac 3 g/L, K_2_HPO_4_ 0.5 g/L, MgSO_4_ 0.2 g/L, and FeSO_4_·7H_2_O 0.01 g/L) and Clostridial growth medium (CGM) medium (glucose 50 g/L, yeast extract 10 g/L, aspartic amide 2 g/L, (NH_4_)_2_SO_4_ 2 g/L, KH_2_PO_4_ 0.75 g/L, K_2_HPO_4_ 0.75 g/L, MgSO_4_ 0.4 g/L, FeSO_4_ 0.01 g/L, MnSO_4_ 0.01 g/L, and NaCl 1 g/L), respectively. These media were supplemented with 25 μg/mL erythromycin and *Clostridium* strains were grown at 37 °C in an anaerobic incubator. 

### 4.3. Strain Construction

The sucrose transporter gene *StSUT1* from potato was first cloned into *Clostridium* expression vector pSOS95. The complete open reading frame of *StSUT1* was obtained by PCR from pDR196-StSUT1 using primers SUT-F: 5ʹ-AAAGGAGGGATTAAAATGGAGAATGGTACAAAAAGAGAAGG-3ʹ and SUT-R: 5ʹ-TAACTCTTATTTTTATTATTTAATGGAAAGCCCCATGGCGAC-3ʹ. Linear pSOS95 was amplified by PCR from vector pSOS95 DNA using primers pSOS95-F: 5ʹ-TAAAAATAAGAGTTACCTTAAATGGTAACT-3ʹ and pSOS95-R: 5ʹ-TTTAATCCCTCCTTTTAAATTCTGGATCCT-3ʹ. Using an in-fusion cloning system (TaKaRa Bio, Dalian, China), *StSUT1* was fused to plasmid pSOS95, and the resulting recombinant expression plasmid was designated pSOS95-StSUT1. Plasmids pSOS95 and pSOS95-StSUT1 were introduced into *E. coli* JM109-pAN for methylation to avoid rejection by *Clostridium*, and then methylated plasmids were extracted from strain M109-pAN1 for transformation into *C. beijerinckii* NCIMB 8052 and *C. acetobutylicum* DSM 792. Plasmids were introduced into *Clostridium* strains using electrotransformation technology [39]. The recombinant strains of *Clostridium* were designated CB8053 (*C. beijerinckii* 8052/pSOS95), CB8054 *(C. beijerinckii* 8052/pSOS95-StSUT1), CA793 (*C. acetobutylicum* 792/pSOS95) and CA794 (*C. acetobutylicum* 792/pSOS95-StSUT1).

### 4.4. Real-Time Quantitative PCR Analysis

Total RNA was extracted from *Clostridium* cells using the MiniBEST Universal RNA Extraction Kit (TaKaRa). Reverse transcription was performed using PrimeScript™ RT Master Mix (Perfect Real Time; TaKaRa). The qRT-PCR primers qSUT-F 5ʹ-CCTTGGTCACGCCTCCGGT-3ʹ and qSUT-R 5ʹ-GCATTTGCTGTTCTCATCC-3ʹ were used for the amplification of *StSUT*, and qRT-PCR primers 16srRNA-F 5ʹ-GCTCGTGTCGTGAGATGTT-3ʹ and 16srRNA-R 5ʹ-TGTAGCCCAGGTCATAAGG-3ʹ were used for the amplification of *16S rRNA*. RT-qPCR was performed with TB Green™ Premix Ex Taq™ II (Tli RNaseH Plus) (TaKaRa) on a 7500 Real-Time PCR System (Applied Biosystems, Foster City, CA, USA). The 2^−ΔΔ^Ct method was used to detect relative gene expression levels, and *16S rRNA* was used as the reference gene. Three technical replicates were tested for every sample, and the data represent the mean of three separate experiments ± standard deviation. A t-test was performed to analyze the relative gene expression levels between CA794 and CB8054 using GraphPad Prism 5 (GraphPad Software Inc., San Diego, CA, USA). 

### 4.5. Transport Assays

*Clostridium* strains used in this study were cultured in TYA or CGM medium for 12 h. Then, 1 mL of bacterial suspension was collected and centrifuged at 1,200× *g* for 5 min at room temperature. The bacterial pellet was then washed twice with hydrogen disodium phosphate solution (25 mM) at the appropriate pH, and the cells were resuspended in 200 μL of the same buffer containing esculin. The cells were incubated for 2 h at 37 °C in an anaerobic incubator. Then the bacteria were harvested and washed as described above and finally resuspended in 1 mL of hydrogen disodium phosphate solution (25 mM) at the appropriate pH. The bacterial solution was added to a 96-well microtiter plate. Fluorescence was measured using a black enzyme marker plate at 367 nm excitation and 454 nm emission with a Sunrise microplate reader (Tecan Group Ltd., Mannedorf, Switzerland), and the optical density of 600 nm (OD_600_) value was measured using a colorless, transparent enzyme marker plate. The relative fluorescence value was expressed as the fluorescence value per OD unit. Three technical replicates were tested for every sample, and the data represent the means of three separate experiments ± standard deviation.
